# Targeting PCSK9 in Vascular Smooth Muscle Cells: An Effective Strategy to Suppress Ferroptosis and Attenuate Abdominal Aortic Aneurysm Progression

**DOI:** 10.1111/cpr.70244

**Published:** 2026-06-10

**Authors:** Mengdie Xia, Man Li, Yanyu Chen, Jialin Chen, Yuting Cui, Xi‐Long Zheng, Jing Yang, Bingzhao Li, Xiaofeng Ma, Miao Liu, Gang Fan, Juan Peng, Xiaoyan Dai, Zhihan Tang

**Affiliations:** ^1^ Department of Cardiology, Affiliated Nanhua Hospital, Hengyang Medical School University of South China Hengyang China; ^2^ Institute of Cardiovascular Disease, Key Laboratory for Arteriosclerology of Hunan Province, Hunan International Scientific and Technological Cooperation Base of Arteriosclerotic Disease, School of Basic Medical Sciences, Hengyang Medical School University of South China Hunan China; ^3^ Department of Cardiovascular Medicine, the Second Affiliated Hospital University of South China Hunan China; ^4^ Departments of Biochemistry & Molecular Biology and Physiology & Pharmacology, Cumming School of Medicine University of Calgary Calgary Alberta Canada; ^5^ Department of Metabolism and Endocrinology Shenzhen University Sixth Affiliated Hospital, Shenzhen Nanshan People's Hospital Guangdong China; ^6^ Department of Pathology Brigham and Women's Hospital, Harvard Medical School Boston Massachusetts USA; ^7^ Department of Urology Shenzhen University Sixth Affiliated Hospital, Shenzhen Nanshan People's Hospital Guangdong China; ^8^ Clinical Research Institute, the Second Affiliated Hospital University of South China Hunan China; ^9^ School of Basic Medical Sciences Hunan University of Medicine Huaihua China

**Keywords:** abdominal aortic aneurysm, ferroptosis, PCSK9, PROTAC, vascular smooth muscle cells

## Abstract

Abdominal aortic aneurysm (AAA) lacks effective pharmacotherapy. This study examines whether proprotein convertase subtilisin/kexin type 9 (PCSK9) drives ferroptosis in vascular smooth muscle cells (VSMCs) and whether its pharmacological degradation mitigates disease progression. PCSK9 is enriched in VSMCs of human AAA and in murine models induced by PPE or Ang II. SMC‐specific PCSK9 overexpression (*PCSK9* ^SMC OE^) increases aortic diameter, aggravates elastin fragmentation and collagen deposition and elevates MMP2/9 expression. Within aortic lesions, *PCSK9* ^SMC OE^ enhances iron accumulation and lipid peroxidation while reducing glutathione GPX4, consistent with ferroptosis. In primary VSMCs, PCSK9 overexpression suppresses GPX4 and glutathione, increases malondialdehyde and Fe^2+^ levels and impairs viability, whereas PCSK9 knockdown attenuates Ang II–induced ferroptosis. Mechanistically, PCSK9 triggers ferritinophagy, as shown by decreased ferritin heavy chain‐1 (FTH1) and nuclear receptor coactivator‐4 (NCOA4), an increased LC3‐II/I ratio and enhanced FTH1–LAMP1 colocalisation. Autophagy inhibition with bafilomycin A1 blocks Fe^2+^ accumulation and rescues ferroptotic indices. The cell‐permeable peptide Cadd4 promotes PCSK9 degradation, restores FTH1 and NCOA4 and suppresses ferroptosis in VSMCs. In PPE and Ang II models, Cadd4 reduces aortic dilation, preserves medial structure and normalises ferroptosis and ferritinophagy markers. PCSK9 drives ferritinophagy‐dependent ferroptosis in VSMCs, and Cadd4 represents a promising therapeutic strategy for AAA.

## Graphical abstract

PCSK9 aggravates AAA by promoting ferroptosis in VSMCs. In the pathological AAA environment, PCSK9 upregulation triggers ferritinophagy‐dependent degradation of FTH1, leading to Fe^2+^ release, triggering lipid peroxidation and ferroptosis, exacerbating AAA. Pharmacological degradation of PCSK9 with the PROTAC molecule Cadd4 inhibits this pathway, suppresses ferroptosis and attenuates aneurysm development.

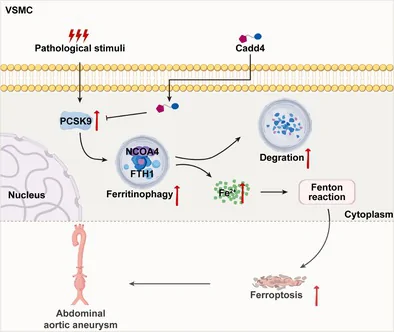

## 1 Introduction

Abdominal aortic aneurysm (AAA) is a life‐threatening vascular disease characterised by progressive dilation of the abdominal aortic wall and formation of an aneurysmal mass [1, 2], with rupture conferring mortality rates approaching 80% [3, 4]. Despite this high clinical burden, no pharmacological interventions are available to prevent or slow AAA progression [1]. Surgical repair, either open or endovascular, remains the only established treatment; however, it is constrained by perioperative morbidity, long‐term complications and imperfect durability [1, 5, 6]. These unmet clinical needs highlight the urgent need to elucidate the molecular mechanisms driving AAA and to identify actionable therapeutic targets.

Vascular smooth muscle cells (VSMCs) are critical for maintaining the structural integrity of the aortic media, and their dysfunction represents a central pathological hallmark of AAA [5, 7, 8]. Accumulating evidence indicates that ferroptosis, an iron‐dependent form of regulated cell death, contributes significantly to VSMC injury and aneurysm progression [9, 10, 11]. However, the key regulators that disrupt intracellular iron homeostasis and trigger VSMC ferroptosis within the aneurysmal wall remain inadequately characterised, representing a pivotal knowledge gap.

Proprotein convertase subtilisin/kexin type 9 (PCSK9) is a well‐established modulator of cholesterol homeostasis that governs plasma low density lipoprotein (LDL)‐cholesterol levels by promoting the lysosomal degradation of hepatocyte LDL receptors, thereby limiting receptor recycling and reducing LDL clearance [12, 13]. In addition to this canonical function, recent experimental studies have implicated PCSK9 in cellular lipid peroxidation [14], processes intimately associated with ferroptosis. Genome‐wide association studies and animal models further indicate that PCSK9 genetic variants or deficiency modulate AAA development in a manner that appears [15, 16, 17], at least partly independent of circulating lipid levels [15], suggesting potential lipid‐independent actions of PCSK9 within the vascular wall.

In this study, we demonstrate that PCSK9 is upregulated in VSMCs from both human AAA specimens and murine AAA models. PCSK9 overexpression facilitates ferritinophagy‐mediated iron release and potentiates VSMC ferroptosis, whereas pharmacological depletion of PCSK9 using the PROTAC degrader Cadd4 suppresses ferroptosis and attenuates aneurysm progression. These findings delineate a lipid‐independent, VSMC‐autonomous role for PCSK9 in AAA pathogenesis and position PCSK9 degradation as a promising therapeutic strategy for AAA.

## 2 Results

### 2.1 PCSK9 Expression Is Enriched in VSMCs of Human and Mouse AAA

To investigate the potential involvement of PCSK9 in AAA pathogenesis, human abdominal aortic specimens were analysed. Immunohistochemical analysis revealed significantly elevated PCSK9 expression in aneurysmal tissues compared to non‐AAA controls (Figure 1A). Immunofluorescence further confirmed that PCSK9 upregulation was predominantly localised to VSMCs, with minimal expression in endothelial cells or macrophages, supporting a VSMC‐enriched expression pattern (Figure 1B). To further validate this observation, we isolated primary mouse aortic VSMCs (MVSMCs), primary mouse aortic endothelial cells (MAECs), primary mouse aortic adventitial fibroblasts (MAAFs) and peritoneal macrophages (PMs) from the same mice, and assessed PCSK9 protein expression. As shown in Figure S1, among the four cell types examined, MVSMCs exhibited the highest level of PCSK9 protein expression, whereas PCSK9 expression was lower in MAECs, MAAFs and PMs. These results indicate that PCSK9 is not exclusively expressed in VSMCs, but is markedly enriched in VSMCs compared with endothelial cells, fibroblasts and macrophages. Consistently, in mouse AAA models induced by PPE or Ang II, PCSK9 immunofluorescence predominantly co‐localised with VSMCs and was markedly upregulated in aneurysmal vessels (Figure 1C,D). Western blotting analysis corroborated elevated PCSK9 protein levels in the aortic medial layers of AAA mice compared to saline‐treated controls (Figure 1E,F). Together, these data demonstrate that PCSK9 is significantly upregulated in both human and murine aneurysmal aortas and is specifically enriched within VSMCs, where it may actively contribute to AAA pathogenesis.

**FIGURE 1:**
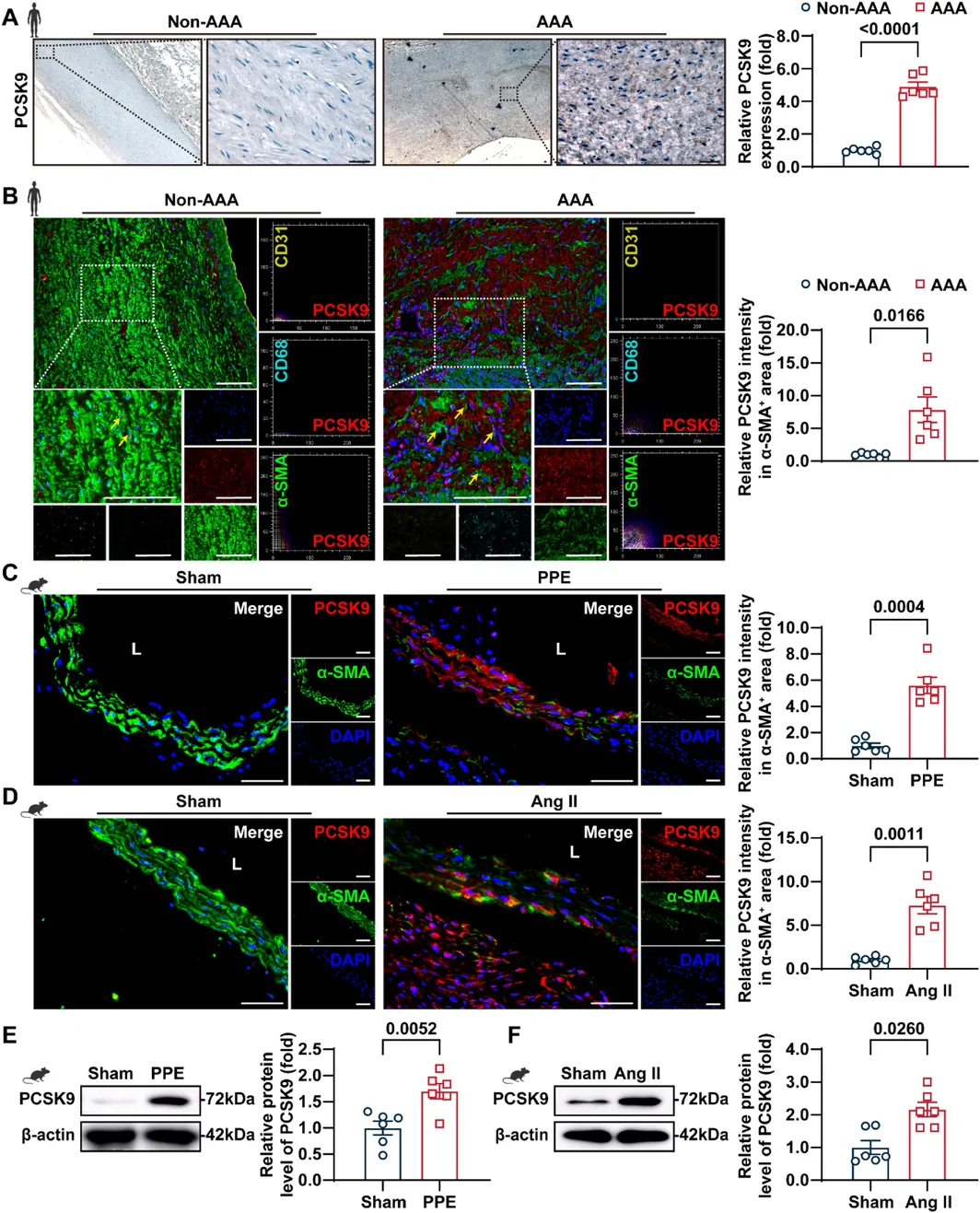
Elevated PCSK9 expression in human and murine AAA aortic tissues. (A) Immunohistochemical images and quantification of PCSK9 in aortic tissues of non‐AAA controls and AAA patients (Scale bar: 100 μm; *n* = 6 per group). (B) Immunofluorescence images showing PCSK9 (red), CD31 (yellow), CD68 (cyan), α‐SMA (green) and DAPI (blue) in aortic tissues of non‐AAA controls and AAA patients (Scale bar: 100 μm; *n* = 6 per group). Colocalisation scatter plots (right) indicate the correlation between PCSK9 and each marker; closer proximity to the diagonal reflects stronger colocalisation. PCSK9 intensity was quantified within α‐SMA^+^ areas. (C) Immunofluorescence of PCSK9 (red), α‐SMA (green) and DAPI (blue) in infrarenal aortas from PPE‐induced mice (Scale bar: 100 μm; *n* = 6 per group), with quantification restricted to α‐SMA^+^ areas. (D) Immunofluorescence of PCSK9 (red), α‐SMA (green) and DAPI (blue) in suprarenal aortas of Ang II‐induced *Apoe* ^−/−^ mice (Scale bar: 100 μm; *n* = 6 per group), with quantification restricted to α‐SMA^+^ areas. (E and F) Western blot and quantification of PCSK9 protein in the medial layer of aortas from PPE‐induced and Ang II‐induced *Apoe* ^−/−^ mice, respectively (*n* = 6 per group). Data are presented as mean ± SEM. Statistical significance was determined using unpaired two‐tailed Student's *t* test or Welch's *t* test. AAA, abdominal aortic aneurysm; Ang II, angiotensin II; non‐AAA, non‐AAA control; PPE, porcine pancreatic elastase.

### 2.2 SMC‐Specific PCSK9 Overexpression Aggravates AAA Progression

To directly evaluate the contribution of VSMC‐derived PCSK9 to AAA development, we generated SMC‐specific PCSK9‐overexpressing mice (*PCSK9* ^SMC OE^) and littermate controls (*PCSK9* ^fl/fl^). Successful recombination was confirmed by PCR‐based genotyping of Tagln‐Cre, Rosa26 wildtype and Rosa26 targeted (*Pcsk9*) alleles (Figure S2A,B). Furthermore, primary VSMCs isolated from *PCSK9* ^SMC OE^ mice exhibited markedly increased PCSK9 mRNA and protein levels compared with control VSMCs, as determined by qPCR and Western blotting, respectively (Figure S2C,D). Consistently, immunofluorescence staining showed increased PCSK9 expression within α‐SMA‐positive areas of the aortic wall (Figure S2E). Following induction of AAA with PPE (Figure 2A), *PCSK9* ^SMC OE^ mice displayed significantly larger maximal aortic diameters compared to controls (Figure 2B,C). Histomorphometric analysis showed well‐preserved elastic lamellae in control aortas, whereas *PCSK9* ^SMC OE^ aortas displayed severe elastic fibre fragmentation and enhanced aberrant collagen deposition within the medial layer (Figure 2D). Immunofluorescence also showed elevated MMP2 and MMP9 expression (Figure 2E,F). These results indicate that SMC‐specific PCSK9 overexpression aggravates AAA progression by promoting medial degeneration and aortic dilation.

**FIGURE 2:**
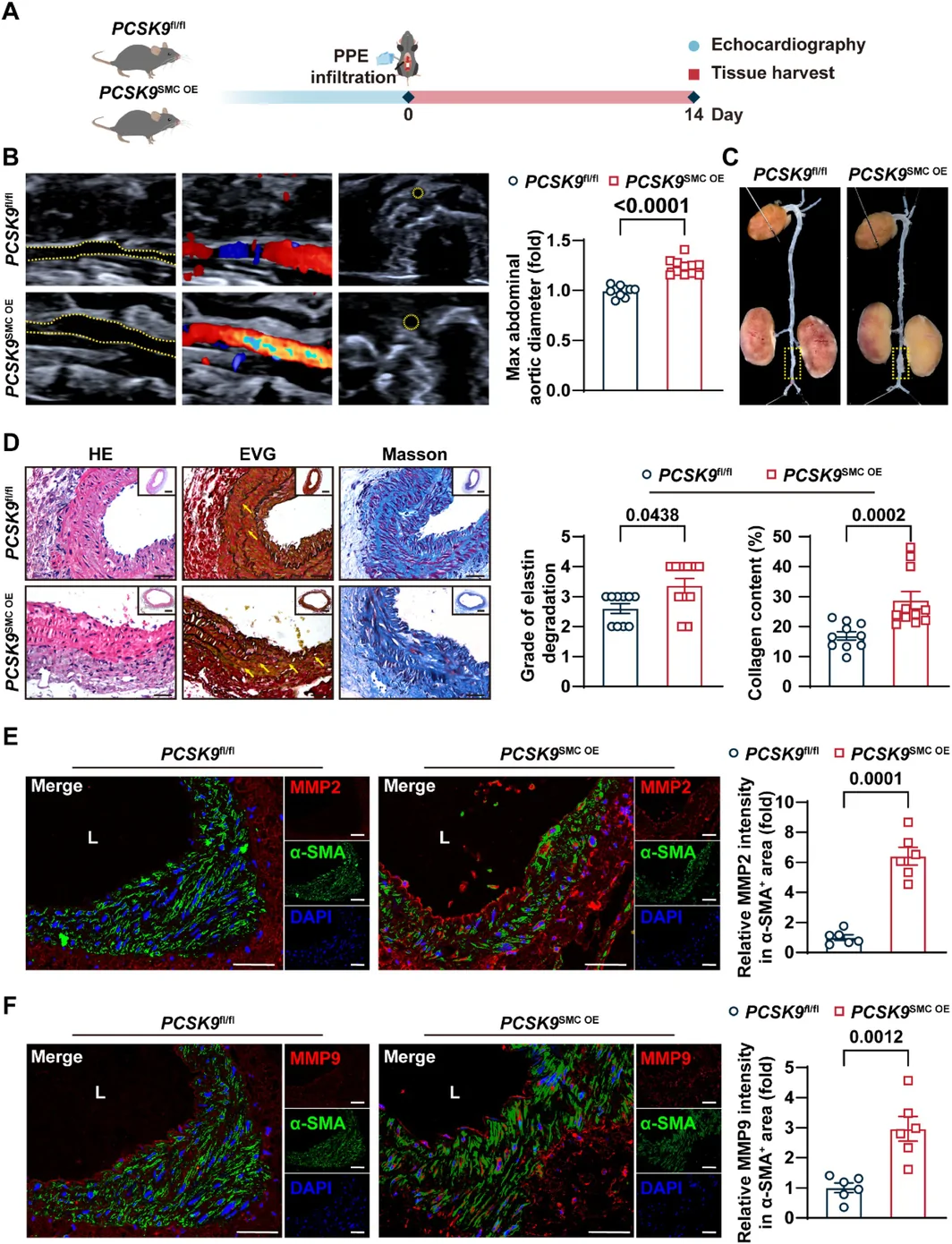
SMC‐specific PCSK9 overexpression exacerbates AAA formation. (A) Experimental timeline for 3 U PPE‐induced AAA in *PCSK9* ^SMC OE^ and *PCSK9* ^fl/fl^ mice. (B) Representative ultrasound images of abdominal aortas, with quantification of maximal infrarenal aortic diameter (*n* = 10–11 per group). (C) Representative gross morphology of abdominal aortas from *PCSK9* ^fl/fl^ and *PCSK9* ^SMC OE^ mice; rectangles indicate aneurysmal regions. (D) Representative H&E, EVG and Masson's trichrome staining of infrarenal aortic sections, with quantification of collagen deposition and elastin degradation grade (Scale bar: 100 μm; *n* = 10–11 per group). (E) Representative immunofluorescence images of MMP2 (red), α‐SMA (green) and DAPI (blue) in infrarenal aortas (Scale bar: 100 μm; *n* = 6 per group). MMP2 intensity was quantified within α‐SMA^+^ areas. (F) Representative immunofluorescence images of MMP9 (red), α‐SMA (green) and DAPI (blue) in infrarenal aortas (Scale bar: 100 μm; *n* = 6 per group). MMP9 intensity was quantified within α‐SMA^+^ areas. Data are presented as mean ± SEM. Statistical significance was determined by unpaired two‐tailed Student's *t* test, Welch's *t* test or Mann–Whitney U test (for ordinal data of elastin degradation grade). EVG, Elastica van Gieson; H&E, haematoxylin and eosin; *PCSK9* ^fl/fl^, littermate control mice; *PCSK9* ^SMC OE^, SMC‐specific PCSK9‐overexpressing.

### 2.3 PCSK9 Induces VSMC Ferroptosis in AAA

Given that SMC‐specific PCSK9 overexpression markedly exacerbated AAA, we next investigated the underlying cellular mechanisms. Primary mouse aortic VSMCs were isolated, infected with Ad‐NC or Ad‐PCSK9 and subjected to transcriptomic profiling (Figure 3A). RNA sequencing identified 474 differentially expressed genes (DEGs) that overlapped with those from human AAA‐associated transcriptional signatures. KEGG pathway enrichment of these intersecting DEGs revealed significant involvement of ferroptosis‐related pathways (Figure 3B,C).

**FIGURE 3:**
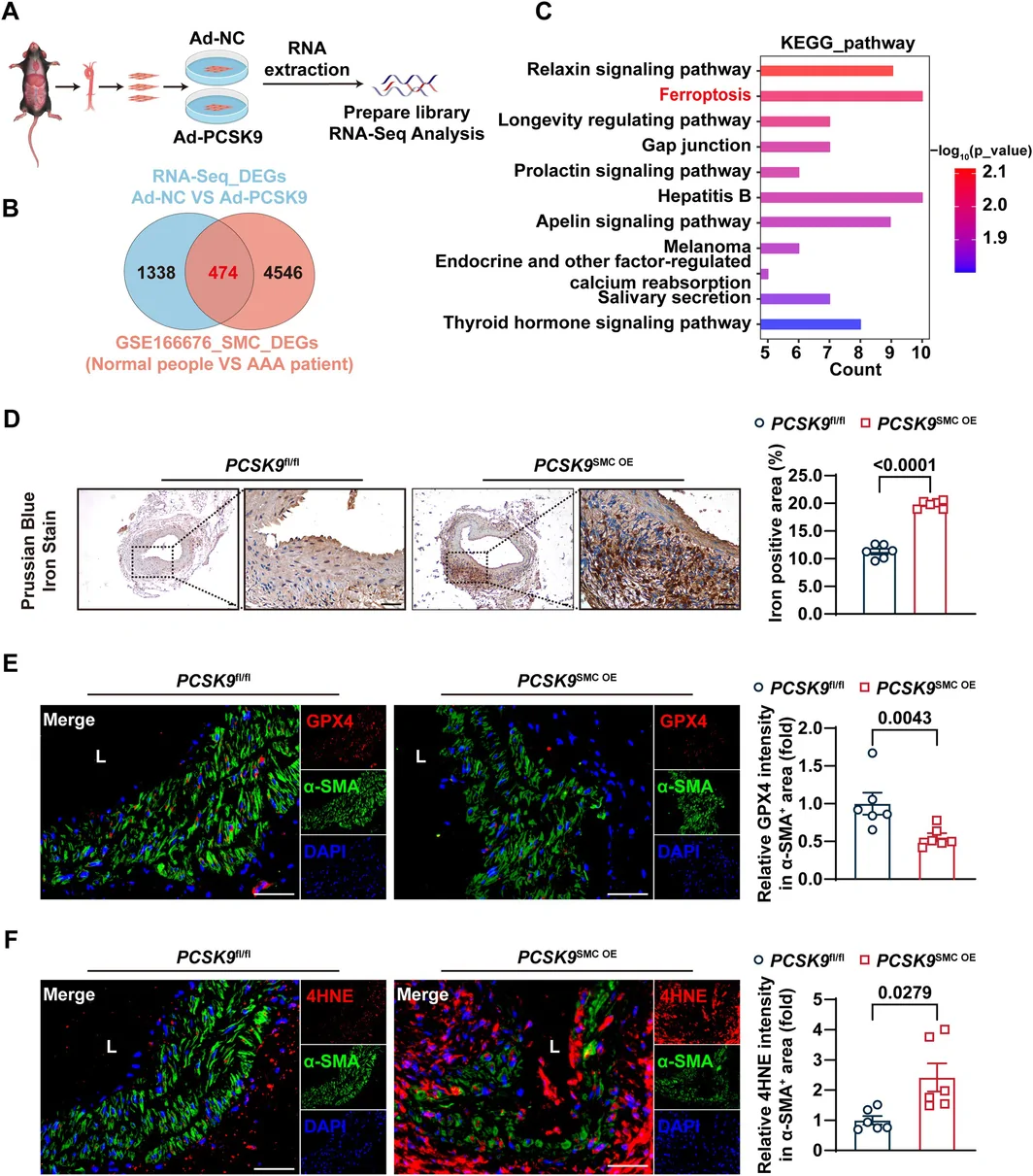
PCSK9‐driven ferroptosis is implicated in both human and murine models. (A) RNA‐seq analysis of primary mouse VSMCs. (B) Venn diagram showing the overlap of differentially expressed genes (DEGs; *p* < 0.05, |logFC| > 1) between PCSK9‐overexpressing primary VSMCs and the human AAA scRNA‐seq dataset GSE166676. (C) KEGG pathway analysis of the intersecting genes. (D) Representative images and quantification of Prussian blue iron staining enhanced with DAB in infrarenal aortas (Scale bar: 100 μm; *n* = 5 per group). (E) Representative immunofluorescence images of GPX4 (red), α‐SMA (green) and DAPI (blue) in infrarenal aortas of PPE‐induced mice (Scale bar: 100 μm; *n* = 6 per group). GPX4 fluorescence was quantified within α‐SMA^+^ areas. (F) Representative immunofluorescence images of 4HNE (red), α‐SMA (green) and DAPI (blue) in infrarenal aortas (Scale bar: 100 μm; *n* = 6 per group). 4HNE fluorescence was quantified within α‐SMA^+^ areas. Data are presented as mean ± SEM. Statistical significance was determined using unpaired two‐tailed Student's *t* test or Welch's *t* test. 4HNE, 4‐hydroxynonenal; GPX4, glutathione peroxidase 4; VSMCs, vascular smooth muscle cells.

In vivo, Prussian blue staining showed enhanced iron deposition in aortic sections of in *PCSK9* ^SMC OE^ mice (Figure 3D). Subsequent immunofluorescence demonstrated reduced expression of glutathione peroxidase 4 (GPX4) and increased accumulation of 4‐hydroxynonenal (4HNE) in α‐SMA^+^ VSMC regions (Figure 3E,F). These findings suggest that VSMC‐derived PCSK9 promotes ferroptosis in AAA.

### 2.4 PCSK9 Contributes to Ang II‐Induced VSMC Ferroptosis

Consistent with in vivo observations, in vitro experiments indicated that PCSK9 overexpression induced characteristic ferroptotic phenotypes, including marked reduction in GPX4 protein levels, depletion of glutathione (GSH), accumulation of malondialdehyde (MDA) and impaired cell viability (Figure S3A,B; Figure 4A–D). To determine whether PCSK9 is necessary for Ang II‐induced ferroptosis, VSMCs were transfected with siPCSK9 prior to Ang II stimulation. PCSK9 knockdown abolished the ferroptotic response, reflected by restored GPX4 and GSH levels, suppressed MDA accumulation and improved cell viability (Figure S3C,D; Figure 4E–H).

**FIGURE 4:**
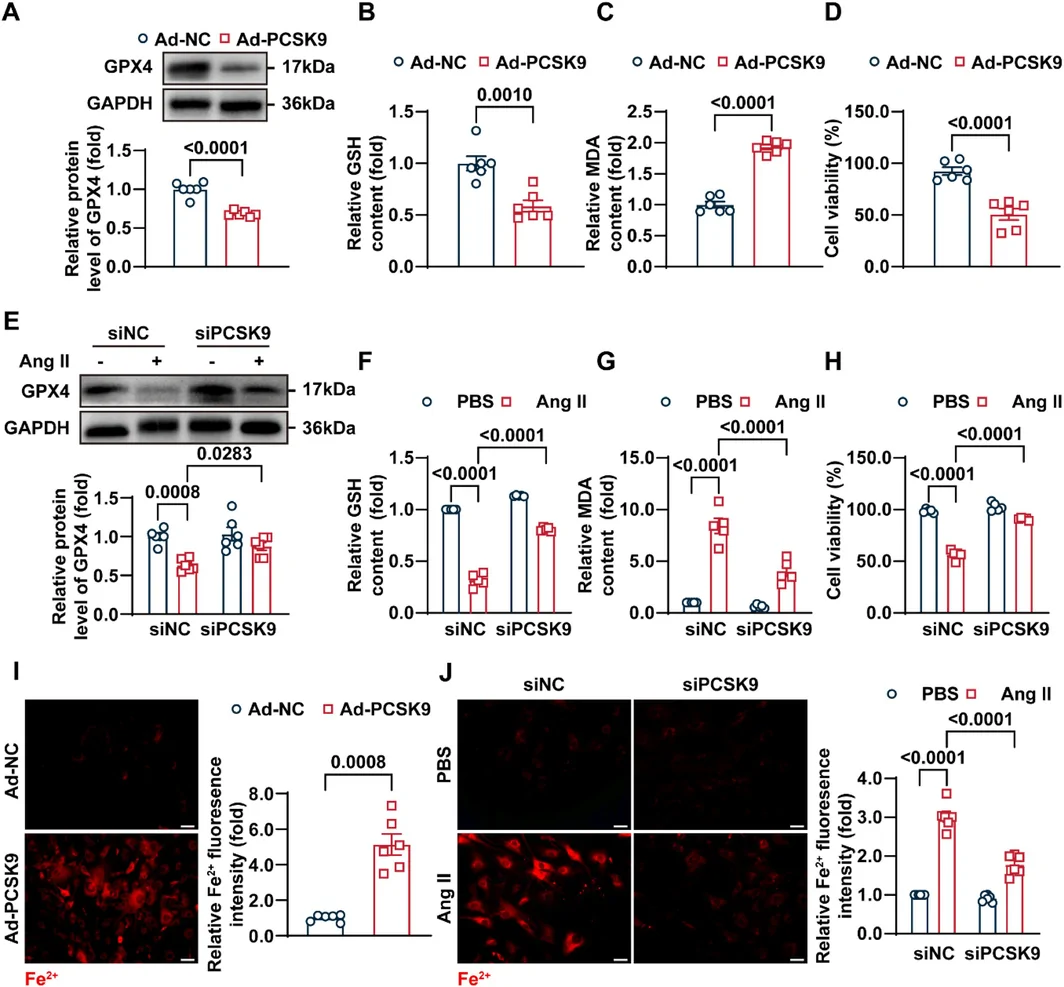
PCSK9 promotes ferroptosis in VSMCs. (A–D) VSMCs were transfected with Ad‐NC or Ad‐PCSK9. (A) Representative Western blot and quantification of GPX4 protein levels (*n* = 6 per group). (B) Quantification of GSH levels (*n* = 6 per group). (C) Quantification of MDA levels (*n* = 6 per group). (D) Quantification of cell viability (*n* = 6 per group). (E–H) VSMCs were transfected with siPCSK9 or siNC, followed by treatment with angiotensin II (Ang II, 2 μM, 24 h). (E) Representative Western blot and quantification of GPX4 protein levels (*n* = 6 per group). (F) Quantification of GSH levels (*n* = 5 per group). (G) Quantification of MDA levels (*n* = 6 per group). (H) Quantification of cell viability (*n* = 5 per group). (I) Representative images and quantitative analysis of FerroOrange staining (Fe^2+^) in VSMCs transfected with Ad‐NC or Ad‐PCSK9 (Scale bar: 50 μm; *n* = 6 per group). (J) Representative images and quantification of FerroOrange staining (Fe^2+^) in siPCSK9‐transfected VSMCs with or without Ang II (Scale bar: 50 μm; *n* = 6 per group). Data are presented as mean ± SEM. Statistical significance was determined using unpaired two‐tailed Student's *t* test or Welch's *t* test (panels A–D, I) or two‐way ANOVA (panels E–H, J). GSH, glutathione; MDA, malondialdehyde.

Since iron overload is a central driver of ferroptosis, intracellular Fe^2+^ levels were measured to evaluate PCSK9‐mediated iron dyshomeostasis. PCSK9 overexpression promoted Fe^2+^ accumulation, whereas PCSK9 knockdown mitigated Ang II‐induced Fe^2+^ elevation (Figure 4I,J). Therefore, PCSK9 drives ferroptosis in VSMCs through dysregulation of iron metabolism.

### 2.5 PCSK9 Promotes Ferritinophagy in VSMCs

To define the molecular mechanisms underlying PCSK9‐mediated ferroptosis in VSMCs, we conducted a comparative proteomic analysis comparing VSMCs infected with Ad‐PCSK9 to Ad‐NC‐treated controls. The resulting differentially expressed proteins were screened against the top 25 ferroptosis‐ and iron metabolism‐associated proteins curated from the GeneCards database, which revealed three overlapping candidates: ferritin heavy chain 1 (FTH1), heme oxygenase 1 (HMOX1) and transferrin receptor (TFRC) (Figure 5A). Of these, FTH1 emerged as a protein of particular interest due to its established role in ferritinophagy—a selective autophagic process wherein nuclear receptor coactivator 4 (NCOA4) mediates FTH1 degradation, leading to iron release and subsequent promotion of ferroptosis [18, 19, 20] Our results showed that overexpression of PCSK9 markedly reduced FTH1 and NCOA4 protein levels, increased the LC3‐II/I ratio and enhanced the co‐localisation of FTH1 with lysosome‐associated membrane protein 1 (LAMP1, a lysosomal marker) (Figure 5B–D).

**FIGURE 5:**
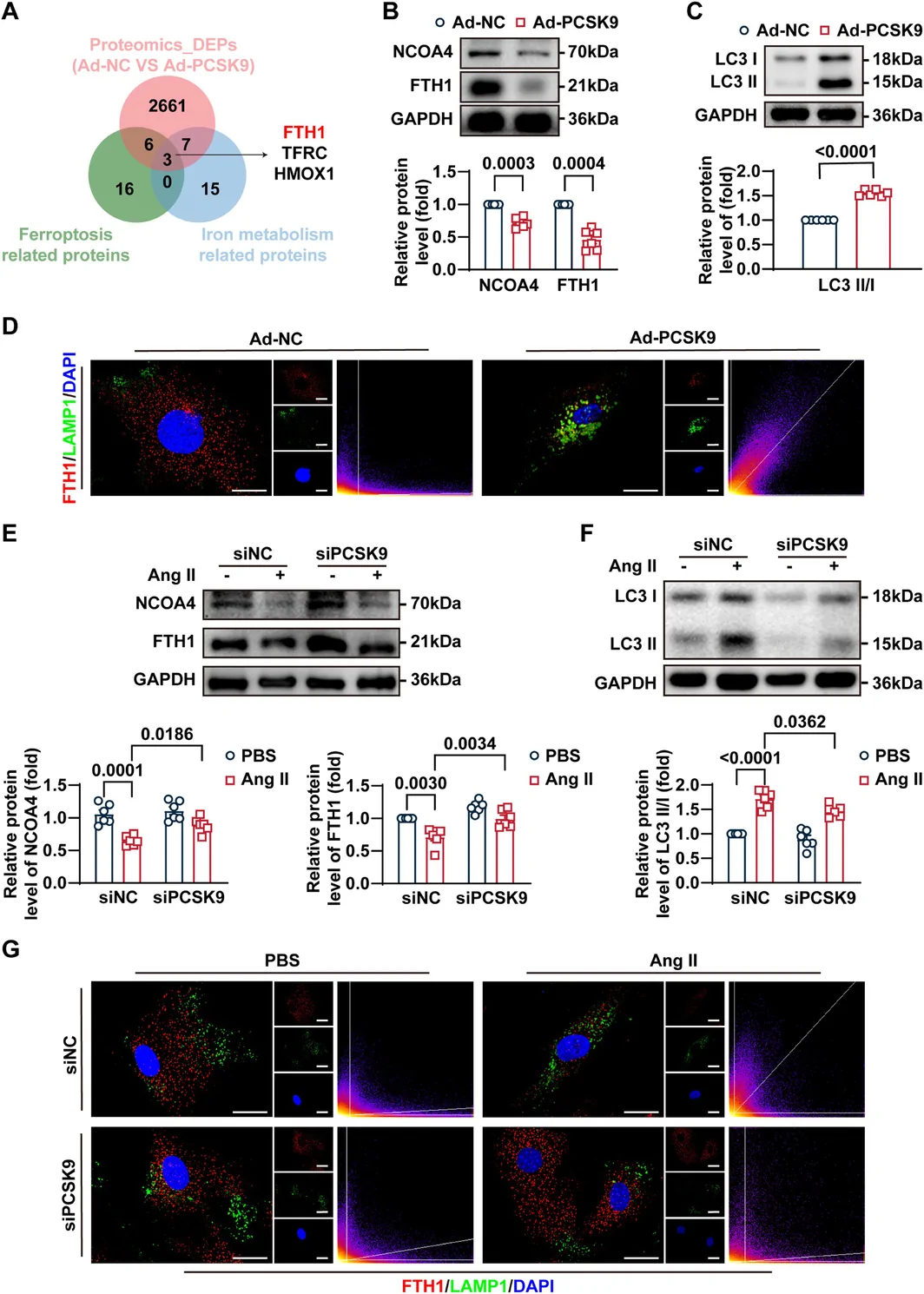
PCSK9 modulates ferritinophagy in VSMCs. (A) Venn diagram showing the overlap between differentially expressed proteins (DEPs) identified by proteomic analysis of primary VSMCs overexpressing PCSK9, the top 25 ferroptosis‐ and iron metabolism‐associated proteins (as retrieved from GeneCards). (B–D) VSMCs were transfected with Ad‐NC or Ad‐PCSK9. (B) Representative Western blot and quantification of NCOA4 and FTH1 protein levels (*n* = 6 per group). (C) Representative Western blot and quantification of LC3 II/I (*n* = 6 per group). (D) Representative immunofluorescence images of FTH1 (red), LAMP1 (green) and DAPI (blue) (Scale bar: 20 μm). Corresponding FTH1/LAMP1 fluorescence‐intensity scatterplots are shown on the right. Increased diagonal clustering indicates enhanced FTH1–LAMP1 colocalisation. (E–G) VSMCs were transfected with siPCSK9 or siNC, followed by treatment with Ang II (2 μM, 24 h). (E) Representative Western blot and quantification of NCOA4 and FTH1 protein levels (*n* = 6 per group). (F) Representative Western blot and quantification of LC3 II/I (*n* = 6 per group). (G) Representative immunofluorescence images of FTH1 (red), LAMP1 (green) and DAPI (blue) (Scale bar: 20 μm). Corresponding FTH1/LAMP1 fluorescence‐intensity scatterplots are shown on the right. Increased diagonal clustering indicates enhanced FTH1–LAMP1 colocalisation. Data are presented as mean ± SEM. Statistical significance was determined using unpaired two‐tailed Student's *t* test or Welch's *t* test (panels B‐D) or two‐way ANOVA (panels E–G). FTH1, ferritin heavy chain 1; NCOA4, nuclear receptor coactivator 4.

Conversely, in VSMCs transfected with siPCSK9 or siNC and stimulated with Ang II, both FTH1 and NCOA4 expression were restored, the LC3‐II/I ratio was elevated and lysosomal trafficking of FTH1 was reduced (Figure 5E–G), suggesting that PCSK9 is essential for Ang II‐induced ferritinophagy.

### 2.6 Blocking Autophagy Abrogates PCSK9‐Induced Ferritinophagy and Ferroptosis in VSMCs

To elucidate the mechanistic contribution of ferritinophagy to PCSK9‐driven ferroptosis, autophagic flux was inhibited using bafilomycin A1 (BafA1) in PCSK9‐overexpressing VSMCs. Autophagy inhibition resulted in increased LC3‐II/I ratios, restored FTH1 and NCOA4 protein expression, and diminished FTH1–LAMP1 co‐localisation, indicating effective suppression of ferritinophagy (Figure 6A–C). Consequently, intracellular Fe^2+^ accumulation was reduced (Figure 6D), accompanied by reversal of ferroptotic markers, including recovered GPX4 expression, restored GSH levels, suppressed MDA accumulation and enhanced cell viability (Figure 6E–H). Collectively, these results indicate that ferritinophagy plays a crucial role in PCSK9‐induced ferroptosis in VSMCs.

**FIGURE 6:**
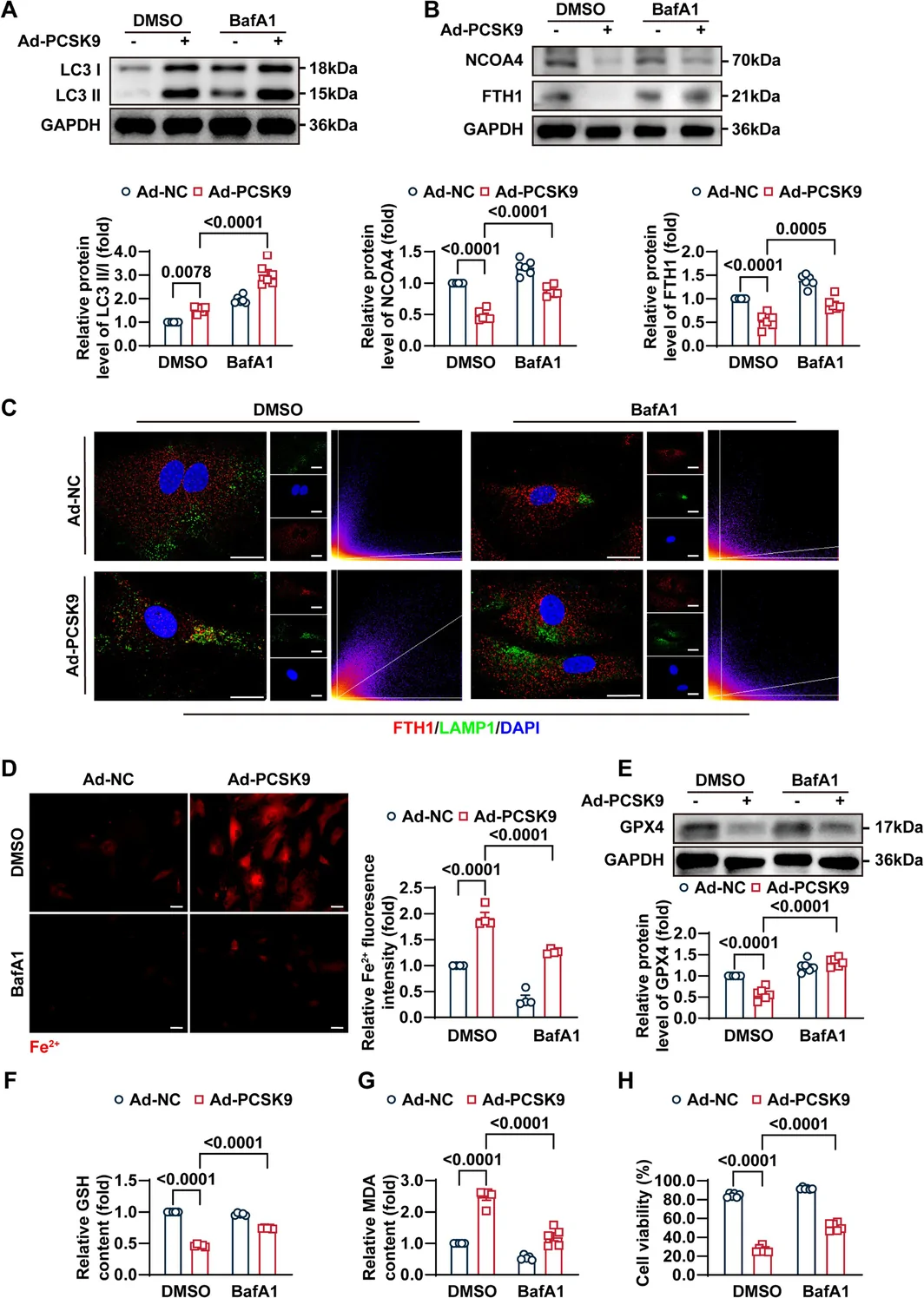
Blocking autophagy rescues PCSK9‐induced ferritinophagy and ferroptosis in VSMCs. (A–H) VSMCs were transfected with Ad‐PCSK9, then treated with or without Bafilomycin A1 (BafA1, an autophagy inhibitor). (A) Representative Western blot and quantification of LC3 II/I (*n* = 6 per group). (B) Representative immunofluorescence images of FTH1 (red), LAMP1 (green) and DAPI (blue) (Scale bar: 20 μm). (C) Representative Western blot and quantification of NCOA4 and FTH1 protein levels (*n* = 6 per group). Corresponding FTH1/LAMP1 fluorescence‐intensity scatterplots are shown on the right. Increased diagonal clustering indicates enhanced FTH1–LAMP1 colocalisation. (D) Representative immunofluorescence images and quantification of FerroOrange staining (Fe^2+^) in VSMCs (Scale bar: 50 μm; *n* = 4 per group). (E) Representative Western blot and quantification of GPX4 protein levels (*n* = 6 per group). (F) Quantitative analysis of GSH levels (*n* = 5 per group). (G) Quantitative analysis of MDA levels (*n* = 5 per group). (H) Quantitative analysis of cell viability (*n* = 6 per group). Data are presented as mean ± SEM. Statistical significance was determined using two‐way ANOVA. BafA1, bafilomycin A1.

### 2.7 PCSK9‐Degrading Peptide Cadd4 Attenuates Ferritinophagy and Ferroptosis In Vitro

To explore the therapeutic potential of targeting PCSK9, we utilised Cadd4 [21], a synthetic peptide designed via computer‐aided drug design to selectively promote ubiquitination and proteasomal degradation of PCSK9. Using its intrinsic fluorescence, we observed that Cadd4 was efficiently internalised by VSMCs in a time‐ and dose‐dependent manner (Figure 7A). Cadd4 treatment led to a dose‐dependent decrease in PCSK9 protein levels, an effect that was abolished by co‐treatment with the proteasome inhibitor MG132 (Figure 7B,C). Additionally, Cadd4 suppressed ferritinophagy, as evidenced by restoration of FTH1 and NCOA4 expression and a decrease in LC3‐II/I ratio under Ang II stimulation (Figure 7D,E). Furthermore, Cadd4 attenuated ferroptosis by reducing intracellular Fe^2+^ accumulation, restoring GPX4 and GSH levels and decreasing MDA content (Figure 7F–I). In summary, these data demonstrate that Cadd4 efficiently penetrates VSMCs and induces degradation of PCSK9, thereby inhibiting ferritinophagy and ferroptosis.

**FIGURE 7:**
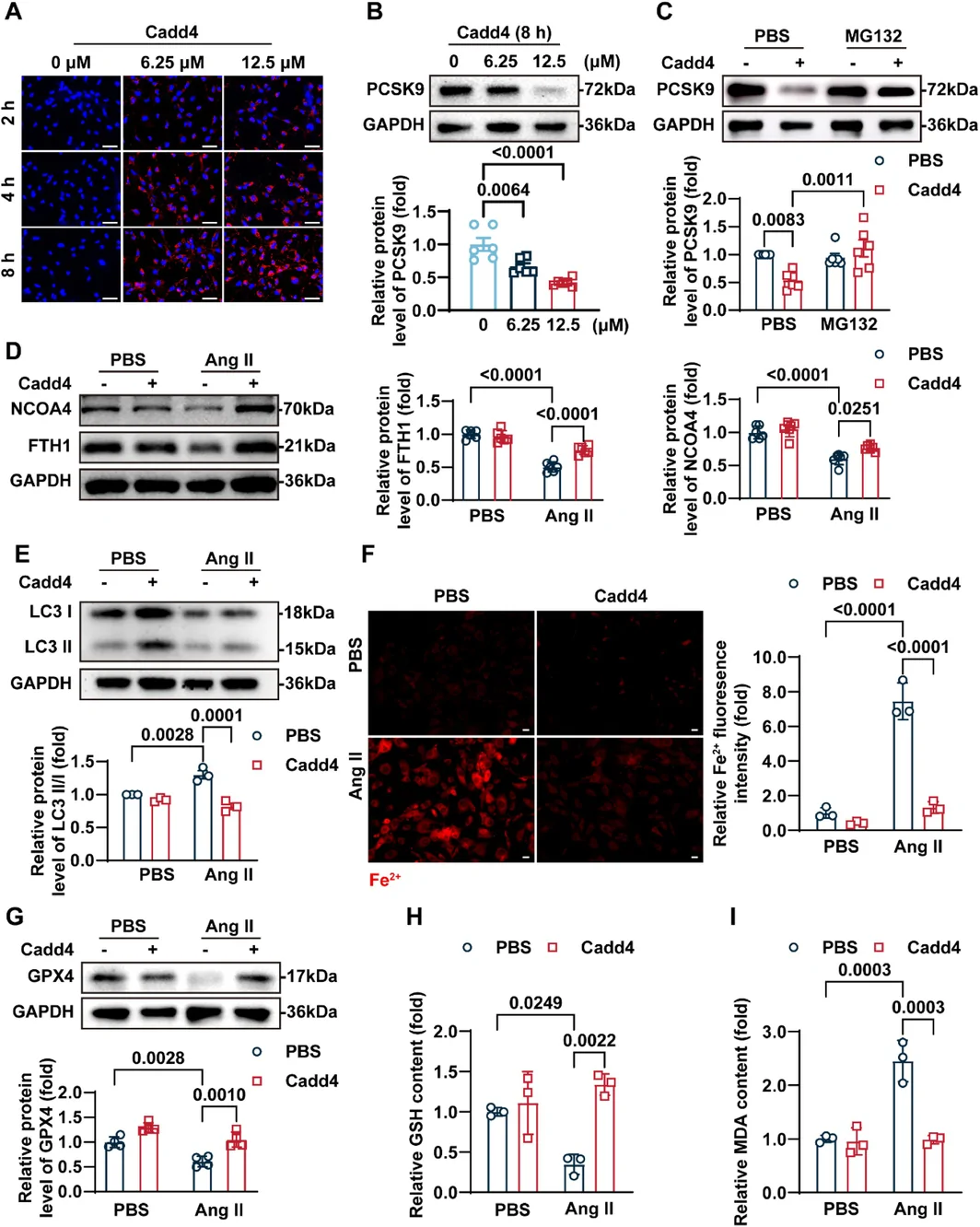
Cadd4 promotes PCSK9 degradation in VSMCs, inhibiting ferritinophagy and ferroptosis. (A) Time‐ and dose‐dependent uptake of fluorescent PROTAC‐Cadd4 (red) in VSMCs (Scale bar: 100 μm). (B) Representative Western blot and quantification of PCSK9 protein levels in VSMCs treated with Cadd4 (*n* = 6 per group). (C) Representative Western blot and quantification of PCSK9 protein levels in VSMCs treated with Cadd4 (12.5 μM, 8 h), with or without the proteasome inhibitor MG132 (10 μM, 6 h) (*n* = 6 per group). (D) Representative Western blot and quantification of NCOA4 and FTH1 protein levels in VSMCs treated with Cadd4, with or without Ang II (*n* = 6 per group). (E) Representative Western blot and quantification of LC3 II/I in VSMCs treated with Cadd4, with or without Ang II (*n* = 3 per group). (F) Representative immunofluorescence images and quantification of FerroOrange staining (Fe^2+^) in VSMCs (Scale bar: 50 μm; *n* = 3 per group). (G) Representative Western blot and quantification of GPX4 protein levels (*n* = 4 per group). (H) Quantification of GSH levels (*n* = 3 per group). (I) Quantification of MDA levels (*n* = 3 per group). Data are presented as mean ± SEM. Statistical significance was determined using one‐way ANOVA (B) or two‐way ANOVA (panels C–I).

### 2.8 Pharmacological Targeting of PCSK9 by Cadd4 Mitigates AAA Progression by Suppressing Ferroptosis and Ferritinophagy

Based on in vitro results, we first confirmed the in vivo efficacy of Cadd4 in degrading PCSK9 within aortic VSMCs. Immunofluorescence analysis of aortic sections from Cadd4‐treated C57BL/6J mice showed reduced PCSK9 expression in α‐SMA^+^ areas, confirming successful PCSK9 degradation (Figure S4).

In a PPE‐induced AAA model (Figure 8A), administration of Cadd4 significantly attenuated aneurysm progression, as indicated by reduced maximal infrarenal aortic diameter, lower AAA incidence and smaller aneurysmal areas (Figure 8B–D). Histological examination revealed preserved elastic laminae and decreased aberrant medial collagen deposition, as demonstrated by H&E, EVG and Masson's trichrome staining (Figure 8E). Moreover, Cadd4 reduced the expression of MMP2 and MMP9 in α‐SMA^+^ VSMCs (Figure 8F). Concurrently, Cadd4 suppressed ferroptosis, evidenced by restored GPX4 levels and decreased 4HNE accumulation in α‐SMA^+^ regions (Figure 8G). Furthermore, Cadd4 inhibited ferritinophagy, reflected by the recovery of FTH1 and NCOA4 protein levels in α‐SMA^+^ VSMCs (Figure 8H).

**FIGURE 8:**
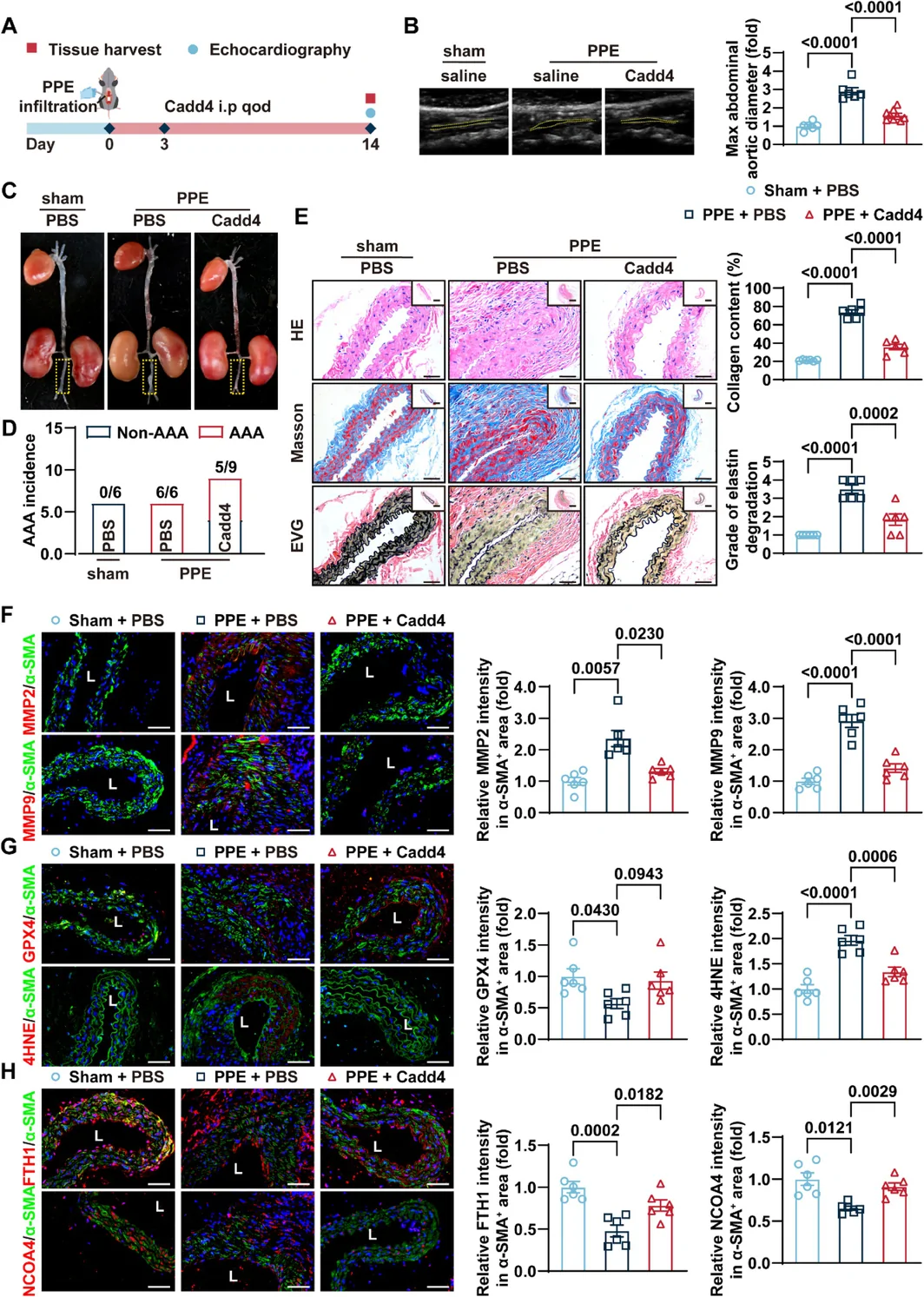
Therapeutic targeting of PCSK9 mitigates PPE‐induced AAA by suppressing ferroptosis and ferritinophagy. (A) Experimental timeline for 3 U PPE‐induced C57BL/6J mice, with or without Cadd4 (20 mg/kg, i.p. every other day). (B) Representative ultrasound of abdominal aortas, with quantification of the maximal infrarenal aortic diameter (*n* = 6 per group). (C) Representative gross morphology of abdominal aortas, rectangles indicate AAA regions. (D) AAA incidence (*n* = 6–9 per group). (E) Representative H&E, EVG and Masson trichrome staining of infrarenal aortic sections, with quantification of collagen deposition and elastin degradation grade (Scale bar: 100 μm; *n* = 6 per group). (F) Representative immunofluorescence images and quantification of MMP2 and MMP9 (red), α‐SMA (green) and DAPI (blue) in infrarenal aortas (Scale bar: 100 μm; *n* = 6 per group). Quantification was performed within α‐SMA^+^ areas. (G) Representative immunofluorescence images and quantitative analysis of GPX4 and 4HNE (red), α‐SMA (green) and DAPI (blue) in infrarenal aortas (Scale bar: 100 μm; *n* = 6 per group). Quantification was performed within α‐SMA^+^ areas. (H) Representative immunofluorescence images and quantitative analysis of NCOA4 and FTH1 (red), α‐SMA (green) and DAPI (blue) in infrarenal aortas (Scale bar: 100 μm; *n* = 6 per group). Quantification was performed within α‐SMA^+^ areas. Data are presented as mean ± SEM. Statistical significance was determined using one‐way ANOVA.

In an Ang II‐induced AAA model, Cadd4 treatment also reduced aortic dilation and AAA incidence (Figure S5A–F) and preserved medial structure, as shown by histological evaluation (Figure S5G). Molecular analyses confirmed decreased MMP2/9 expression and normalisation of ferroptosis and ferritinophagy markers in α‐SMA^+^ VSMCs (Figure S5H–J).

Taken together, these findings demonstrate that pharmacological inhibition of PCSK9 by Cadd4 attenuates VSMC ferroptosis and ferritinophagy in multiple preclinical AAA models, highlighting its potential as a novel therapeutic strategy.

## 3 Discussion

AAA continues to pose a significant clinical challenge due to the lack of effective pharmacological interventions. In this study, we demonstrate that PCSK9 is markedly enriched in VSMCs derived from both human and murine AAA tissues. SMC‐specific overexpression of PCSK9 exacerbates aneurysm formation, whereas its pharmacological degradation via a novel peptide‐based inhibitor, Cadd4, effectively attenuates aneurysm progression. Mechanistically, PCSK9 facilitates the autophagic degradation of FTH1, resulting in the release of redox‐active iron (Fe^2+^) and promoting ferroptosis in VSMCs. Collectively, our findings demonstrate that targeting PCSK9 in VSMCs effectively suppresses ferroptosis, thereby proposing a novel cell‐intrinsic therapeutic strategy for AAA.

The well‐established role of PCSK9 in cardiovascular disease primarily revolves around its canonical function in which hepatically secreted PCSK9 induces degradation of LDL receptors in hepatocytes, leading to elevated plasma lipid levels and exacerbation of atherosclerosis [13, 22]. Recent genome‐wide association studies and Mendelian randomisation analyses have further associated lipid metabolic pathways with AAA susceptibility, identifying the PCSK9 locus as a significant risk variant [16]. Experimental evidence from murine models supports these findings; hepatic overexpression of PCSK9 accelerates aneurysm formation [15], whereas systemic deficiency attenuates disease progression [17]. However, neutralisation of circulating PCSK9 exerts minimal effects on aneurysm diameter [15] and PCSK9 deletion attenuated AAA formation independently of cholesterol modulation [17]. These data argue against a purely lipid‐driven mechanism and underscore the importance of cholesterol‐independent pathways. We provide the first direct evidence that VSMC‐specific PCSK9 overexpression aggravates aneurysmal pathology, underscoring a VSMC‐autonomous role of PCSK9 in AAA progression.

Accumulating evidence indicates a critical role of ferroptosis in AAA pathogenesis, with studies showing that pharmacological inhibition of ferroptosis attenuates the disease [10, 11, 23] Notably, we observed that PCSK9 overexpression induces characteristic ferroptotic alterations in VSMCs, including intracellular iron accumulation, compromised antioxidant defence (reduced GPX4 and GSH) and elevated lipid peroxidation (increased MDA), whereas PCSK9 knockdown counteracts these effects both in vivo and in vitro. Our findings support the notion that PCSK9 promotes AAA by inducing VSMC ferroptosis. More importantly, we reveal that PCSK9 overexpression activates ferritinophagy, a selective autophagic process mediating FTH1 degradation and subsequent release of labile Fe^2+^, thereby sensitising VSMCs to ferroptosis and accelerating AAA progression. Conversely, PCSK9 reduction attenuates ferritinophagy under AAA‐relevant pathological conditions. Although prior reports suggest that PCSK9 may promote ferroptosis in hepatocellular carcinoma by disrupting antioxidant mechanisms [14], our results establish PCSK9 as a key regulator of ferritinophagy‐driven ferroptosis in VSMCs. This mechanistic insight uncovers a previously unrecognised pathway connecting PCSK9 to dysregulated iron metabolism and AAA development.

Although PCSK9 is traditionally considered a secreted protein, it is synthesised intracellularly and transits through the secretory pathway before release. The precise mechanisms through which PCSK9 regulates ferritinophagy remain unclear. Several studies support the idea that PCSK9 may modulate ferritinophagy through the activation of upstream signalling pathways. Specifically, PCSK9 has been shown to activate the p38 MAPK pathway [24, 25, 26]. Importantly, p38 MAPK signalling has been demonstrated to promote NCOA4‐dependent ferritinophagy [27, 28] Based on these observations, we propose a working model in which PCSK9 activates the p38 MAPK pathway to induce NCOA4‐dependent ferritinophagy. However, it is crucial to note that the direct molecular interactions between PCSK9 and the autophagic machinery remain to be experimentally verified.

Currently, surgical intervention is reserved for patients with advanced, symptomatic or high‐risk aneurysms and no effective pharmacotherapies exist for AAA [5, 29]. Most AAAs are detected at a small, asymptomatic stage, precluding surgical eligibility. Moreover, even minimally invasive approaches such as endovascular aortic repair (EVAR) are associated with procedural risks and long‐term complications [30, 31]. Systemic therapies targeting hypertension or dyslipidemia provide limited benefit [32, 33], and emerging interventions that modulate inflammation or vascular remodelling remain confined to the preclinical stage [34, 35, 36]. These limitations highlight the urgent need for therapeutic strategies that target cell‐intrinsic mechanisms within the vascular wall. Building on our finding that VSMC‐derived PCSK9 drives AAA through ferroptosis, we developed Cadd4—a PROTAC designed to catalytically degrade intracellular PCSK9 [21]. PROTAC technology has demonstrated promise across diverse fields, including oncology, viral infection and neurodegenerative disease, highlighting its translational feasibility [37, 38, 39]. In murine AAA models, Cadd4 effectively attenuated aneurysm formation, preserved aortic architecture and restored ferroptosis‐related markers, indicating protection against VSMC ferroptotic injury. Its catalytic mode of action, favourable tissue distribution and scalable synthesis further bolster its translational appeal, establishing targeted degradation of VSMC‐derived PCSK9 as a promising therapeutic avenue.

This work also has limitations that warrant further investigation. Although our findings robustly demonstrate that PCSK9 promotes ferroptosis via ferritinophagy, the upstream mechanisms by which PCSK9 regulates ferritinophagy remain unclear. Previous studies offer potential insights: one reported that the ROS‐ATM‐LKB1‐AMPK axis contributes to PCSK9‐induced autophagy [40], while another highlighted the essential role of the AMPK‐ULK1 pathway in ferritinophagy [41]. Together, these observations suggest that PCSK9 may modulate ferritinophagy through AMPK‐related signalling pathways, warranting further investigation to elucidate the precise molecular mechanisms. Additionally, a comprehensive assessment of the long‐term safety and bioavailability of Cadd4 in large‐animal models is necessary to advance toward clinical translation. Future developments in oral or nanoparticle‐mediated delivery systems may further enhance its therapeutic applicability.

In conclusion, our study establishes PCSK9 as a key driver of AAA via ferritinophagy‐dependent iron release and ferroptosis in VSMCs. By disrupting intracellular iron homeostasis, PCSK9 promotes VSMC ferroptosis and fuels aneurysm progression. Therapeutic targeting of this pathway with Cadd4, a peptide‐based PCSK9 degrader, offers a novel strategy to maintain vascular integrity and inhibit aneurysm expansion. Overall, our findings delineate the PCSK9–ferritinophagy–ferroptosis axis in VSMCs as a mechanistically grounded and therapeutically targetable pathway in AAA.

## 4 Conclusions

AAA is limited to surgical intervention, as no pharmacological therapy has yet demonstrated efficacy in meaningfully altering disease progression. Our findings delineate a cell‐intrinsic signalling axis linking PCSK9, ferritinophagy and ferroptosis in VSMCs and establish that targeted degradation of intracellular PCSK9 using the peptide Cadd4 attenuates aneurysmal expansion and preserves medial layer integrity in preclinical models. Collectively, these results provide a foundation for clinical development of PCSK9‐targeted therapeutics—notably intracellular degraders that act synergistically with extracellular neutralisation approaches—offering a promising strategy for patients with small or inoperable AAAs.

## 5 Experimental Section/Methods

### 5.1 Human AAA Study

Human AAA specimens were obtained from patients undergoing surgical repair at the Department of Pathology, The First Affiliated Hospital of University of South China. Normal aortic control tissues were collected from autopsy cases provided by the Forensic Identification Centre of the same university. All procedures were approved by the Ethics Committee of the University of South China (2024/No.0099), and written informed consent was obtained from all participants or their legal representatives. The investigation conformed to the principles outlined in the Declaration of Helsinki.

### 5.2 Animals

SMC‐specific PCSK9 overexpression (*PCSK9* ^SMC OE^) mice were generated on a C57BL/6J background using a Cre‐loxP system. *PCSK9* ^fl/fl^ mice carrying a Rosa26‐loxP‐stop‐loxP‐mPCSK9 knock‐in allele were crossed with SM22α‐Cre transgenic mice. Offspring positive for SM22α‐Cre were designated as *PCSK9* ^SMC OE^, while Cre‐negative littermates served as controls.

Animal Models of PPE‐Induced AAA: *PCSK9* ^fl/fl^, *PCSK9* ^SMC OE^ and C57BL/6J mice were anaesthetised, and the infrarenal abdominal aorta was exposed and isolated. Each aortic segment was wrapped with sterile bibulous paper soaked in 3 U PPE (LS002292, Worthington) in saline for 30 min. Controls received saline‐soaked paper. After removal, the abdominal cavity was irrigated with saline and sutured [42].

#### 5.2.1 Animal Models of Ang II‐Induced AAA

An Ang II mini‐pump model was established following previously reported methods [23]. Eight‐week‐old male *Apoe* ^−/−^ mice were implanted subcutaneously with osmotic minipumps (1004w, RWD) delivering saline or Ang II (1000 ng/kg/min, 4,006,473, Bachem) for 28 days. Mice were anaesthetised, and a small incision was made in the posterior neck region to insert the pump. The incision was closed with sutures. All mice were fed a high‐fat diet (D12108C, Research Diets) [43].

#### 5.2.2 In Vivo Cadd4 Administration

Mice were administered intraperitoneal injections of Cadd4 (20 mg/kg) or an isotype control every other day, starting on Day 3 postsurgery [21].

#### 5.2.3 Aortic Harvest and Measurements

At the experimental endpoint, abdominal aortic diameters were measured by ultrasound (VINNO 6, Vinno Corporation; S6, SonoScape). All mice were perfused transcardially with 4% paraformaldehyde, and the aortas were carefully dissected, photographed and processed for further analyses. All procedures were conducted in accordance with institutional guidelines and the NIH Guide for the Care and Use of Laboratory Animals. For euthanasia, mice were deeply anaesthetised with isoflurane (2%–2.5%, R510‐22‐10, RWD) until loss of pedal reflexes, followed by cervical dislocation to ensure death.

### 5.3 Histological Studies

Aortic tissues were fixed in 4% paraformaldehyde, paraffin‐embedded and sectioned at 4 μm. Sections were stained with Haematoxylin and Eosin (H&E, G1120, Solarbio), Elastica van Gieson (EVG, G11597, Solarbio) and Masson's trichrome (G1346, Solarbio) according to manufacturers' instructions, and images were captured [44, 45].

### 5.4 Immunohistochemistry (IHC) and Immunofluorescence

Paraffin sections were baked at 65°C for 1 h, deparaffinised in xylene, rehydrated and subjected to microwave antigen retrieval in sodium citrate buffer for 5 min.

For IHC, endogenous peroxidase was quenched with 3% H_2_O_2_ for 10 min, followed by blocking with 10% BSA at 37°C for 30 min. Sections were incubated overnight at 4°C with anti‐PCSK9 (1:100, MA5‐32843, Invitrogen), followed by HRP‐conjugated secondary antibodies (Maixin Biotech KIT‐9710) and DAB visualisation. Nuclei were counterstained with haematoxylin.

For multiplexed immunofluorescence, sequential primary antibody staining was performed using antibodies against PCSK9 (1:100, Ab315480, Abcam), α‐SMA (1:100, 67,735–1‐Ig, Proteintech), CD68 (1:100, MA5‐13324, Invitrogen) and CD31 (1:100, 25–0311‐82, Invitrogen). Staining was developed with the TSA 6‐colour kit (AFIHC026, AiFang Biological) according to the manufacturer's protocol, followed by nuclear counterstaining with DAPI. For conventional immunofluorescence, sections were blocked with 10% BSA for 30 min at 37°C and incubated overnight at 4°C with primary antibodies against PCSK9 and α‐SMA (same as above), as well as GPX4 (1:100, ab125066, Abcam), 4HNE (1:100, bs‐6313R, BIOSS), FTH1 (1:100, BM4487, BOSTER), NCOA4 (1:100, 10,968–1‐AP, Proteintech), MMP2 (1:100, 10,373–2‐AP, Proteintech) and MMP9 (1:100, 110,375–2‐AP, Proteintech). After washing, sections were incubated with appropriate secondary antibodies for 1 h at room temperature and mounted with DAPI‐containing antifade medium.

### 5.5 Primary VSMC Isolation, Culture and Treatments

Mouse aortic VSMCs were isolated by enzymatic digestion with elastase (0.744 U/mL, Worthington), collagenase II (1 mg/mL, Worthington) and trypsin inhibitor (1 mg/mL, Worthington) in DMEM/F12. After removal of the adventitia, cells were seeded in DMEM/F12 containing 20% FBS for 7 days, then maintained in 10% FBS with 1% penicillin–streptomycin at 37°C under 5% CO_2_. To simulate pathological conditions, VSMCs were treated with Ang II (2 μM, Bachem) for 24 h, and where indicated, autophagy was inhibited with bafilomycin A1 (100 nM, 6 h pretreatment) or proteasomal degradation was blocked using MG132 (10 μM). For genetic manipulation, cells were transduced with PCSK9‐overexpressing adenovirus or empty vector control (MOI = 50, 48 h; WZ Biosciences), and siRNA‐mediated knockdown was performed using gene‐specific or negative control siRNA (150 nM) with the riboFECT CP Transfection Kit (Ribobio) according to the manufacturer's instructions.

### 5.6 Western Blot

Cells or tissues were lysed in RIPA buffer with protease inhibitors, centrifuged and protein concentrations measured by BCA assay (#23227, ThermoFisher). Equal protein amounts were separated by SDS‐PAGE, transferred onto PVDF membranes and blocked with 5% skim milk. Membranes were incubated overnight at 4°C with primary antibodies: anti‐GAPDH (1:5000, 10494‐1‐AP, Proteintech), anti‐PCSK9 (1:1000, 55206‐1‐AP, Proteintech), anti‐GPX4 (1:1000, A11243, ABclonal), anti‐FTH1 (1:1000, BM4487, BOSTER) and anti‐LC3 (1:1000, 14600‐1‐AP, Proteintech). HRP‐conjugated secondary antibodies were applied for 1.5 h at room temperature. Detection was performed using ECL, and densitometric analysis was done with ImageJ software.

### 5.7 Cellular Functional Assays

Intracellular ferrous iron was detected by FerroOrange staining, with cells incubated in 1.5 μM FerroOrange (F374, Dojindo) for 30 min at 37°C and fluorescence recorded at Ex/Em = 543/580 nm. Glutathione levels were quantified using the glutathione reductase recycling assay (A006‐2‐1, Nanjing Jiancheng Bioengineering Institute) following total protein measurement by BCA assay (#23227, ThermoFisher), with results normalised to protein content. MDA levels were measured using a microscale MDA assay kit (thiobarbituric acid method, A003‐2‐2, Nanjing Jiancheng Bioengineering Institute) according to the manufacturer's instructions, with absorbance recorded at 532 nm. Cell viability was evaluated by CCK‐8 assay (AWC0114, Abiowell), in which cells seeded in 96‐well plates were incubated with 10% reagent for 2 h at 37°C before absorbance measurement at 450 nm.

### 5.8 Statistical Analysis

Statistical Analysis: Experiments were repeated at least three times independently. Data are presented as mean ± SEM. Two‐group comparisons used unpaired two‐tailed Student's *t* test or Welch's *t* test, and multiple‐group comparisons used one‐way or two‐way ANOVA with appropriate post hoc tests. Analyses were performed with GraphPad Prism 10.1.2.

## Author Contributions

Mengdie Xia, Man Li and Yanyu Chen performing the experiments, analysing the data and drafting the manuscript. Jialin Chen and Yuting Cui assisted with animal experiments and pathological analyses. Xi‐Long Zheng, Jing Yang, Xiaofeng Ma and Bingzhao Li provided advice on experimental design and critically revised the manuscript. Zhihan Tang, Xiaoyan Dai, Juan Peng and Gang Fan conceived and supervised the study, provided overall project management, guided the experimental design, reviewed and edited the manuscript and acquired funding. All authors have read and approved the final version of the manuscript.

## Funding

This work was supported by the Science and Technology Program of Hunan Province (R2023046, 2025JJ90128, 2025JJ90126) and the National Natural Science Foundation of China (82571829, 82570537).

## Conflicts of Interest

The authors declare no conflicts of interest.

## Supporting information


**Figure S1:** PCSK9 expression in primary aortic cell types.
**Figure S2:**. Identification of VSMC‐specific PCSK9‐overexpressing mice.
**Figure S3:**. Modulation of PCSK9 expression in VSMCs.
**Figure S4:**. Cadd4 degrades PCSK9 in vivo.
**Figure S5:**. Therapeutic targeting of PCSK9 mitigates Ang II‐induced AAA by suppressing ferroptosis and ferritinophagy.

## Data Availability

The data that support the findings of this study are available from the corresponding author upon reasonable request.
