# Supplementary material for: Targeting PCSK9 in Vascular Smooth Muscle Cells: An Effective Strategy to Suppress Ferroptosis and Attenuate Abdominal Aortic Aneurysm Progression
Source: Cell Prolif. 2026 Jun 10:e70244. Online ahead of print. doi: 10.1111/cpr.70244 (PMC13326032; doi:10.1111/cpr.70244)
Supplement: Supplementary file 1 — Figure S1: PCSK9 expression in primary aortic cell types. Figure S2:. Identification of VSMC‐specific PCSK9‐overexpressing mice. Figure S3:. Modulation of PCSK9 expression in VSMCs. Figure S4:. Cadd4 degrades PCSK9 in vivo. Figure S5:. Therapeutic targeting of PCSK9 mitigates Ang II‐induced AAA by suppressing ferroptosis and ferritinophagy. [file CPR-9999-e70244-s001.docx]

**Supplementary Material**

**Integrating multi-omics approaches reveals PCSK9-driven ferroptosis in vascular smooth muscle cells as a therapeutic target for abdominal aortic aneurysm**

*Mengdie Xia, Man Li, Yanyu Chen, Jialin Chen, Yuting Cui, Xi-Long Zheng, Jing Yang, Bingzhao Li, Xiaofeng Ma, Miao Liu, Gang Fan*, Juan Peng*, Xiaoyan Dai*, Zhihan Tang**

**Content:**

**Experimental section**

**Figure S1.** PCSK9 expression in primary aortic cell types.

**Figure S2.** Identification of VSMC-specific PCSK9-overexpressing mice.

**Figure S3.** Modulation of PCSK9 expression in VSMCs.

**Figure S4.** Cadd4 degrades PCSK9 in vivo.

**Figure S5.** Therapeutic targeting of PCSK9 mitigates Ang II-induced AAA by suppressing ferroptosis and ferritinophagy.


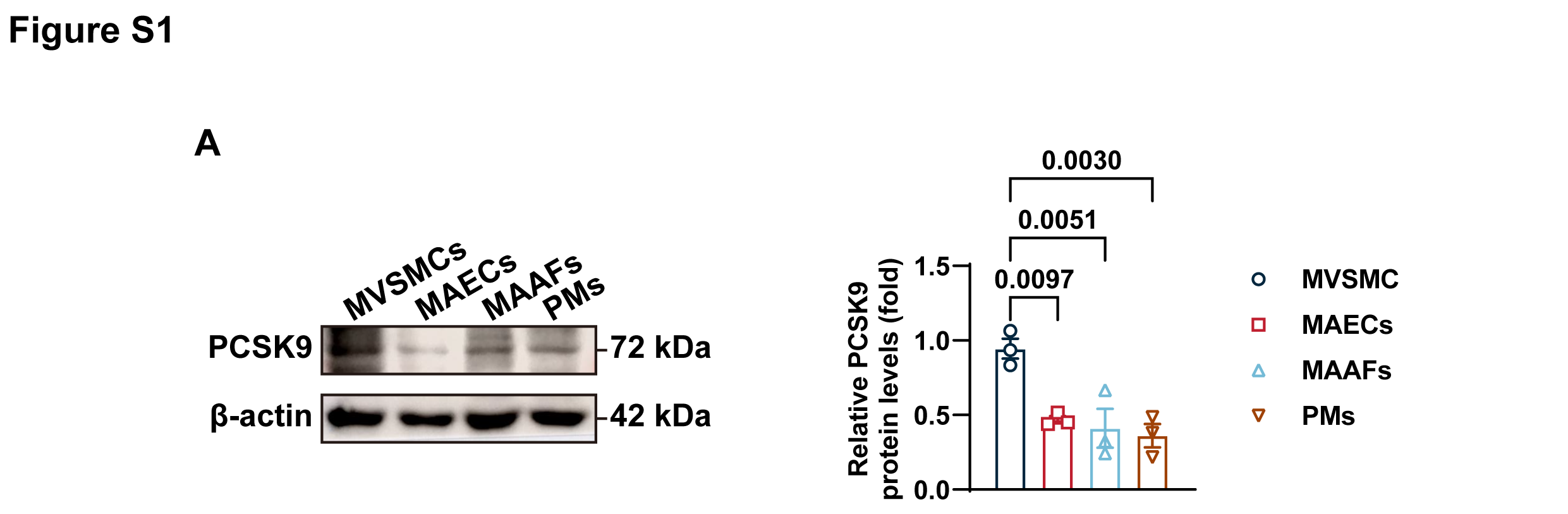


**Figure S1. PCSK9 expression in primary aortic cell types.**

A) Representative Western blot and quantification of PCSK9 in primary mouse aortic VSMCs (MVSMCs), primary mouse aortic endothelial cells (MAECs), primary mouse aortic adventitial fibroblasts (MAAFs), and peritoneal macrophages (PMs) isolated from the same mice (n = 3 per group). Data are presented as mean ± SEM. Statistical significance was determined using one‑way ANOVA.


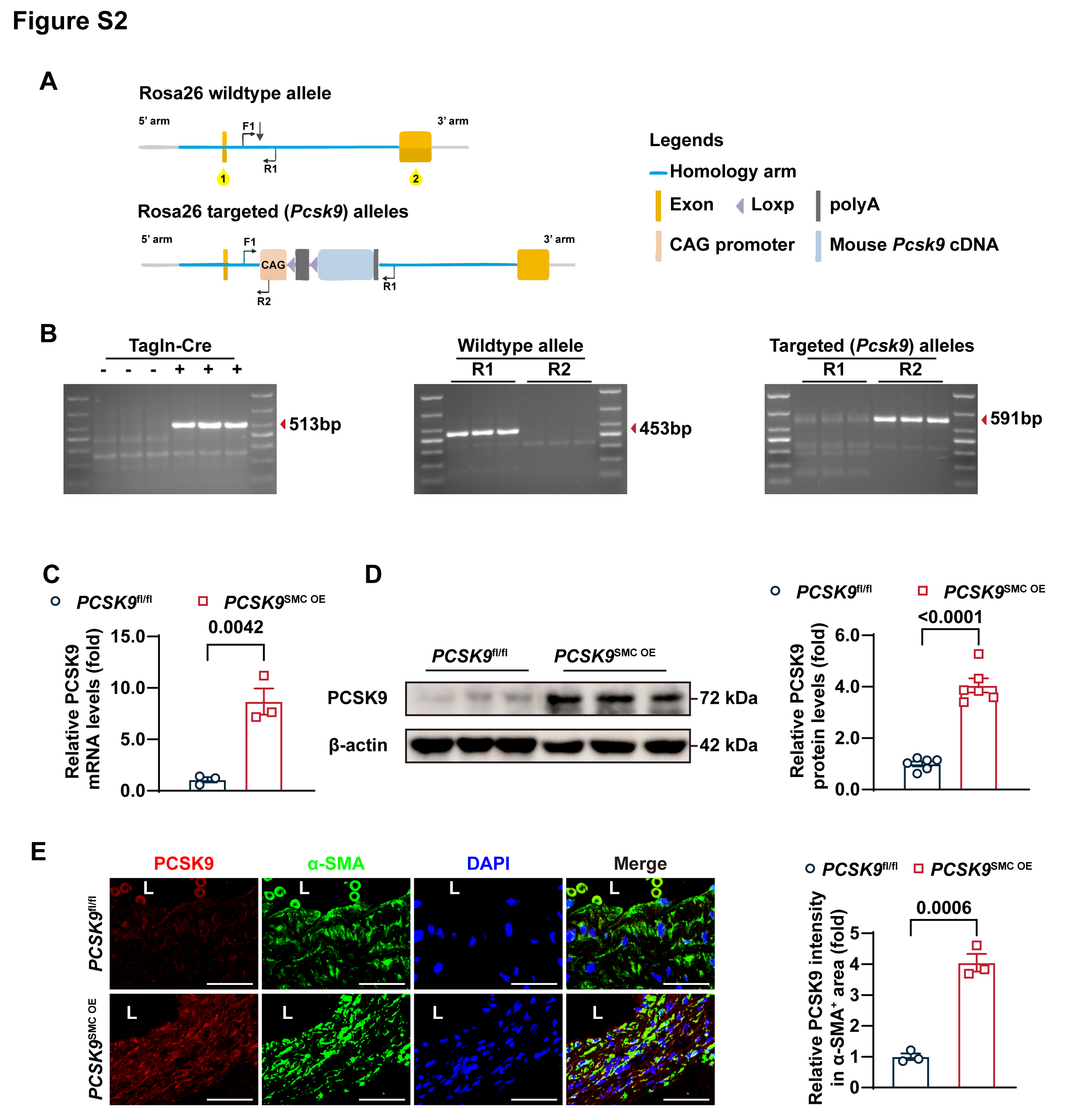


**Figure S2. Identification and validation of SMC-specific PCSK9-overexpressing mice.**

A) Schematic strategy for generating SMC-specific PCSK9-overexpressing mice. B) Representative PCR-based genotyping assays using tail DNA, confirming the presence of Tagln-Cre, Rosa26 wildtype, and Rosa26 targeted (*Pcsk9*) alleles. C) qPCR analysis of PCSK9 mRNA levels in primary VSMCs isolated from the aortas of control and *PCSK9*^SMC OE^ mice (n = 3 per group). D) Representative Western blot and quantification of PCSK9 protein levels in primary VSMCs from control and *PCSK9*^SMC OE^ mice (n = 6 per group). E) Representative immunofluorescence images of PCSK9 (red), α-SMA (green), and DAPI (blue) in aortas from control and *PCSK9*^SMC OE^ mice. Scale bar: 25 μm; n = 3 per group. PCSK9 fluorescence intensity was quantified within α-SMA-positive areas. Data are presented as mean ± SEM. Statistical significance was determined using unpaired two-tailed Student’s *t*-test.


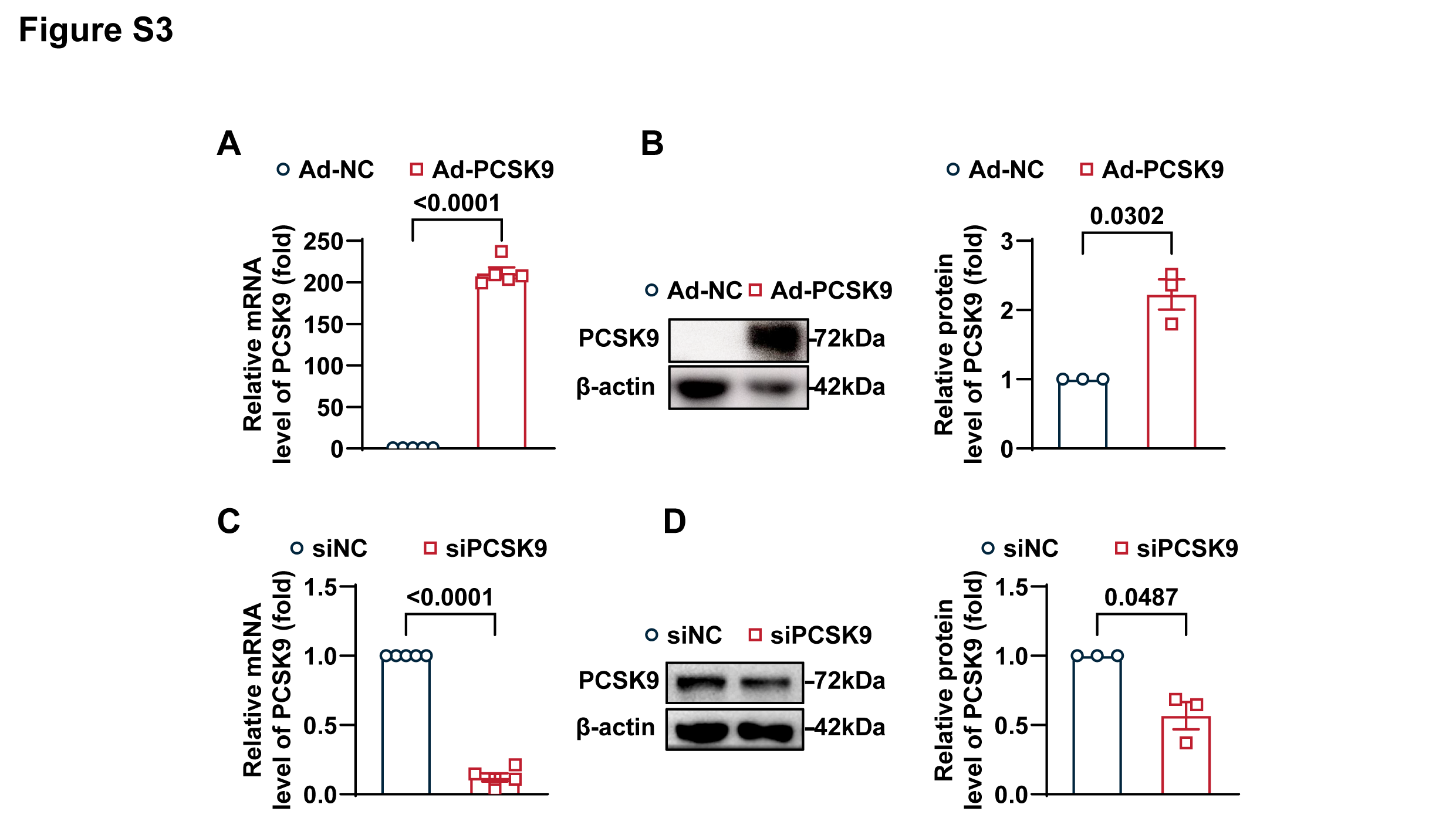


**Figure S2. Modulation of PCSK9 expression in VSMCs.**

A and B) VSMCs were infected with Ad-NC or Ad-PCSK9. A) Quantification of PCSK9 mRNA levels (n = 5 per group). B) Representative Western blot and quantification of PCSK9 protein levels (n = 3 per group). C and D) VSMCs were transfected with siPCSK9 or siNC. C)  Quantification of PCSK9 mRNA level (n = 5 per group). (D) Representative Western blot and quantification of PCSK9 protein levels (n = 3 per group). Data are presented as mean ± SEM. Statistical significance was determined using unpaired two-tailed Student’s *t*-test or Welch’s *t*-test.


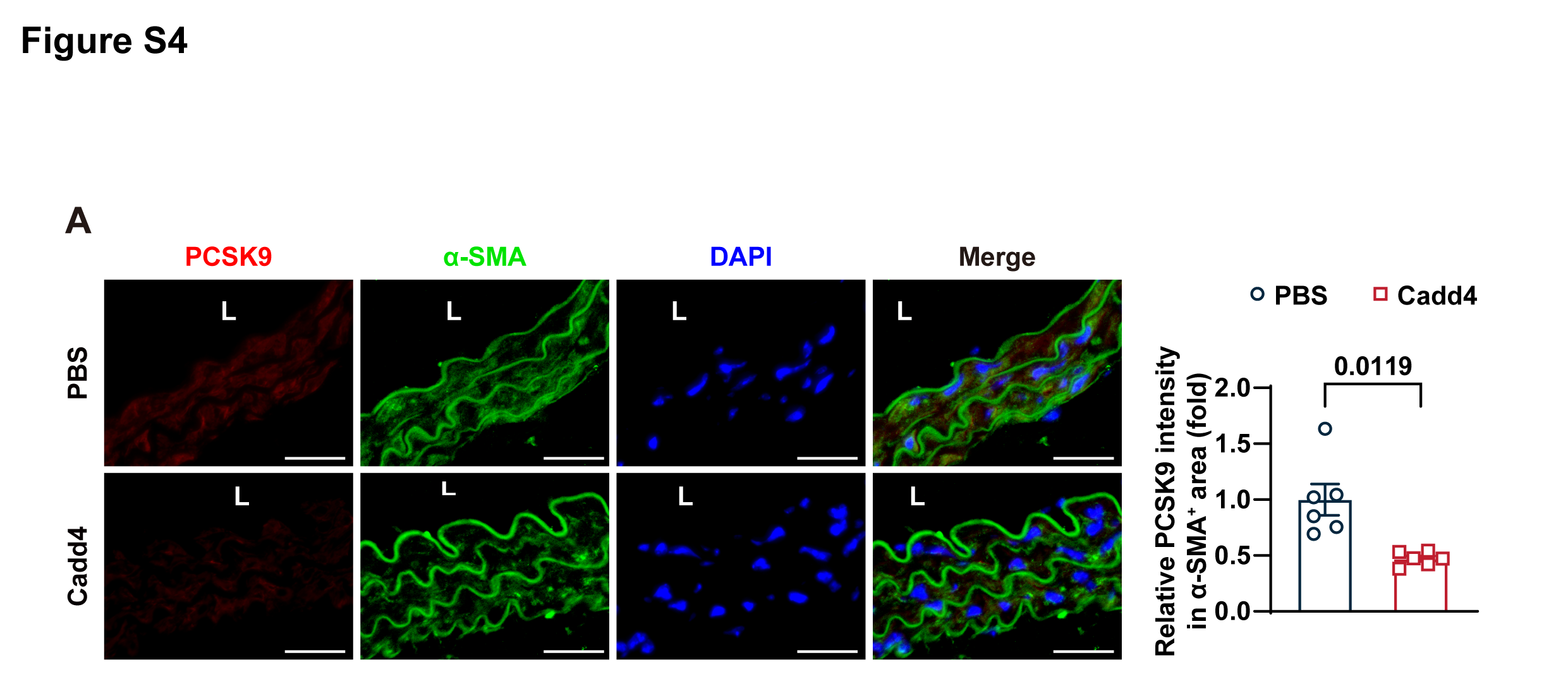


**Figure S4. Cadd4 degrades PCSK9 *in* *vivo*.**

Representative immunofluorescence images and quantification of PCSK9 (red) and DAPI (blue) in aortic sections from C57BL/6J mice treated with or without PROTAC-Cadd4 (20 mg/kg, i.p. every other day; Scale bar: 25 μm; n = 3 per group). PCSK9 fluorescence was quantified within α-SMA⁺ areas. Data are presented as mean ± SEM. Statistical significance was determined using an unpaired two-tailed Student’s *t*-test or Welch’s *t*-test.


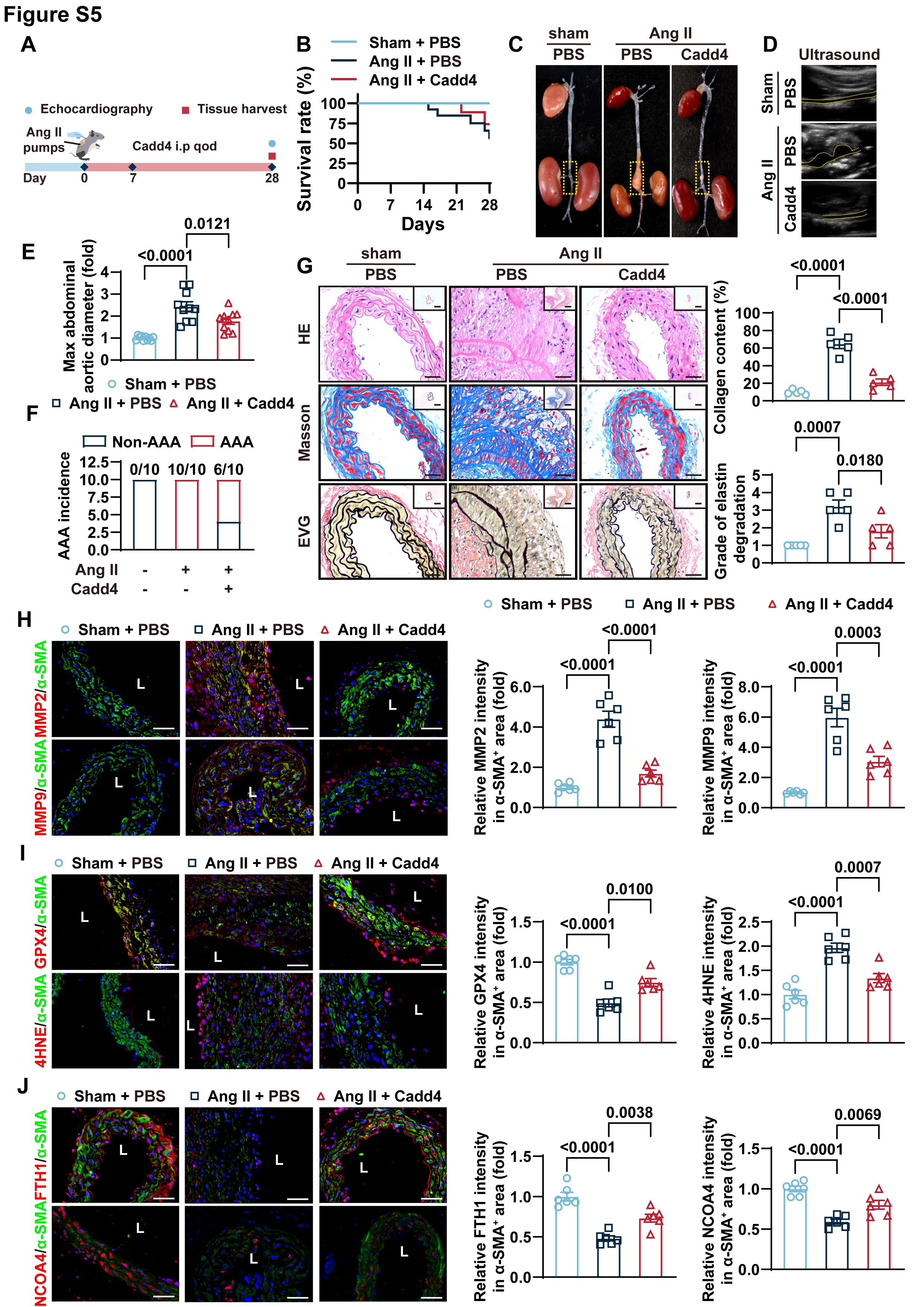


**Figure S5. Therapeutic targeting of PCSK9 mitigates Ang Ⅱ-induced AAA by suppressing ferroptosis and ferritinophagy.**

A) Experimental timeline for Ang Ⅱ-induced AAA in *Apoe*⁻/⁻ mice with or without Cadd4 (20 mg/kg, i.p., every other day). B) Kaplan‑Meier survival curves for the indicated group (n = 10-17 per group). C) Representative gross morphology of abdominal aortas; rectangles indicate AAA regions. D) Representative ultrasound of abdominal aortas. E) The maximal suprarenal aortic diameter (n = 10 per group). F) AAA incidence (n = 10 per group). G) Representative images of HE, EVG, and Masson trichrome staining in suprarenal aortic sections, with quantification of collagen deposition and elastin degradation grade (Scale bar: 100 μm; n = 5 per group). H) Representative immunofluorescence images and quantification of MMP2 and MMP9 (red), α-SMA (green), and DAPI (blue) in suprarenal aortas (Scale bar: 100 μm; n = 6 per group). Quantification was performed within α-SMA⁺ areas. I) Representative immunofluorescence images and quantitative analysis of GPX4 and 4HNE (red), α-SMA (green), and DAPI (blue) in suprarenal aortas (Scale bar: 100 μm; n = 6 per group). Quantification was performed within α-SMA⁺ areas. J) Representative immunofluorescence images and quantitative analysis of NCOA4 and FTH1 (red), α-SMA (green), and DAPI (blue) in suprarenal aortas (Scale bar: 100 μm; n = 6 per group). Quantification was performed within α-SMA⁺ areas. Data are presented as mean ± SEM. Statistical significance was determined using one-way ANOVA.
